# Supplementary material for: Gatekeepers in the health financing scheme: Assessment of knowledge, attitude, practices, and participation of Malaysian private general practitioners in the PeKa B40 scheme
Source: PLoS One. 2023 Oct 17;18(10):e0292516. doi: 10.1371/journal.pone.0292516 (PMC10581488; doi:10.1371/journal.pone.0292516)
Supplement: S1 Table — This table contains the various questionnaires and the responses for each as according to the 5-point Likert scale. (PDF) [file pone.0292516.s001.pdf]

S1 Table Knowledge of health financing schemes (N=296) This table contains the various questionnaires and the responses for each as according to the 5-point Likert scale.

No	Item	n (%)				
		Strongly disagree	Disagree	Neutral	Agree	Strongly agree
1	Tax-based finance refers to finance resources derived from government revenues	6 (2.0)	11 (3.7)	93 (31.4)	106 (35.8)	80 (27.0)
2	Income tax is one revenue source for tax-based finance	2 (0.7)	4 (1.4)	55 (18.6)	123 (41.6)	112 (37.8)
3	Tax-based finance allows risk-sharing between the rich and the poor	7 (2.4)	21 (7.1)	93 (31.4)	102 (34.5)	73 (24.7)
4	Tax-based finance allows healthcare access to each and every citizen	7 (2.4)	22 (7.4)	72 (24.3)	112 (37.8)	83 (28.0)
5	Tax-based finance may lead to underfunding	11 (3.7)	30 (10.1)	129 (43.6)	74 (25.0)	52 (17.6)
6	Progressive taxation is an equitable form of direct taxation in tax-based finance.	3 (1.0)	8 (2.7)	158 (53.4)	87 (29.4)	40 (13.5)
7	The financial resources in Social Health Insurance (SHI) is derived from compulsory contributions from a particular sector.	14 (4.7)	13 (4.4)	87 (29.4)	133 (44.9)	49 (16.6)
8	SHI systems are heavily dependent on payroll contributions	1 (0.3)	10 (3.4)	97 (32.8)	132 (44.6)	56 (18.9)
9	In SHI, payment models can be either single or multi-payor systems	2 (0.7)	5 (1.7)	99 (33.4)	132 (44.6)	58 (19.6)
10	Supervisory institutions are essential in a SHI system to monitor the funding bodies.	1 (0.3)	1 (0.3)	68 (23.0)	136 (45.9)	90 (30.4)
11	SHI systems can ensure equity of access to healthcare.	8 (2.7)	12 (4.1)	91 (30.7)	115 (38.9)	70 (23.6)
12	The 1CARE system proposed by the Ministry of Health in 2010 included SHI as a component.	6 (2.0)	8 (2.7)	146 (49.3)	97 (32.8)	39 (13.2)
13	SHI systems are more costly than tax-based systems to manage	3 (1.0)	23 (7.8)	172 (58.1)	67 (22.6)	31 (10.5)
14	The revenue base in SHI is usually smaller than tax-based finance	5 (1.7)	25 (8.4)	190 (64.2)	46 (15.5)	30 (10.1)
15	SHI is not appropriate in a large informal sector	8 (2.7)	31 (10.5)	167 (56.4)	57 (19.3)	33 (11.1)
16	Contributions to SHI are compulsory.	7 (2.4)	25 (8.4)	111 (37.5)	93 (31.4)	60 (20.3)
17	Private insurance refers to insurance plans from the private sector as	2 (0.7)	12 (4.1)	65 (22.0)	126 (42.6)	91 (30.7)

No	Item	n (%)				
		**Strongly disagree**	**Disagree**	**Neutral**	**Agree**	**Strongly agree**
	opposed to government-funded health finances.					
18	Private insurance is classified as complementary if the coverage is for services excluded by the state	7 (2.4)	8 (2.7)	94 (31.8)	115 (38.9)	72 (24.3)
19	19. Private insurance is classified as supplementary if the coverage is for faster access to healthcare.	5 (1.7)	10 (3.4)	73 (24.7)	127 (42.9)	81 (27.4)
20	Unregulated private insurance can lead to unethical practices such as profiteering.	1 (0.3)	8 (2.7)	43 (14.5)	82 (27.7)	162 (54.7)
21	The existence of a private supplemental insurance can lead to unwarranted referrals.	5 (1.7)	24 (8.1)	57 (19.3)	125 (42.2)	85 (28.7)
22	Private insurance may have a biased preference for clients with good risks known as "cream-skimming" .	0	9 (3.0)	74 (25.0)	113 (38.2)	100 (33.8)
23	Private insurance is based on ability to pay	2 (0.7)	7 (2.4)	35 (11.8)	115 (38.9)	137 (46.3)
24	Private insurance is not the preferred choice for Universal Health Coverage	5 (1.7)	24 (8.1)	96 (32.4)	86 (29.1)	85 (28.7)
