# Supplementary material for: Gatekeepers in the health financing scheme: Assessment of knowledge, attitude, practices, and participation of Malaysian private general practitioners in the PeKa B40 scheme
Source: PLoS One. 2023 Oct 17;18(10):e0292516. doi: 10.1371/journal.pone.0292516 (PMC10581488; doi:10.1371/journal.pone.0292516)
Supplement: S2 Table — The contents of this table focus on the responses to each aspect of the gatekeeper role as according to the 5-point Likert scale. (PDF) [file pone.0292516.s002.pdf]

S2 Table Knowledge of gatekeeper roles (N=296) The contents of this table focus on the responses to each aspect of the gatekeeper role as according to the 5-point Likert scale.

**No**	**Item**	**n (%)**				
		**Strongly disagree**	**Disagree**	**Neutral**	**Agree**	**Strongly agree**
1	A GP gatekeeper triages the patient's further access to the health system	1 (0.3)	2 (0.7)	20 (6.8)	114 (38.5)	159 (53.7)
2	Gatekeeping systems can be either a full system whereby all patients must first be seen by a GP; or partial whereby patients are allowed to bypass the GP and be seen by the secondary specialist directly.	18 (6.1)	26 (8.8)	60 (20.3)	108 (36.5)	84 (28.4)
3	Gatekeeping improves the health system efficiency	1 (0.3)	3 (1.0)	25 (8.4)	113 (38.2)	154 (52.0)
4	Gatekeeping enhances good coordination of patient care.	0	3 (1.0)	22 (7.4)	115 (38.9)	156 (52.7)
5	Gatekeeping reduces waiting times for specialist treatment	1 (0.3)	12 (4.1)	44 (14.9)	108 (36.5)	131 (44.3)
6	Effective gatekeeping requires a good referral system between private GPs and hospital specialists	0	0	14 (4.7)	94 (31.8)	188 (63.5)
7	Gatekeeping promotes quaternary prevention i.e. protect patients from unnecessary and harmful medical interventions	0	3 (1.0)	39 (13.2)	111 (37.5)	143 (48.3)
8	Gatekeeping may cause delayed diagnosis of cancers	68 (23.0)	87 (29.4)	84 (28.4)	45 (15.2)	12 (4.1)
