# Supplementary material for: Gatekeepers in the health financing scheme: Assessment of knowledge, attitude, practices, and participation of Malaysian private general practitioners in the PeKa B40 scheme
Source: PLoS One. 2023 Oct 17;18(10):e0292516. doi: 10.1371/journal.pone.0292516 (PMC10581488; doi:10.1371/journal.pone.0292516)
Supplement: S3 Table — This table indicates the responses according to the 5-point Likert scale on various aspects of the PeKa B40 scheme. (PDF) [file pone.0292516.s003.pdf]

S3 Table Knowledge of PeKa B40 (N=296) This table indicates the responses according to the 5-point Likert scale on various aspects of the PeKa B40 scheme.

No	Item	n (%)				
		Strongly disagree	Disagree	Neutral	Agree	Strongly agree
1	PeKa B40 is a pilot public-private partnership programme.	6 (2.0)	7 (2.4)	73 (24.7)	115 (38.9)	95 (32.1)
2	In PeKa B40 program, GPs are involved only in screening and early detection of non-communicable diseases.	8 (2.7)	13 (4.4)	40 (13.5)	134(45.30)	101 (34.1)
3	One of the primary objectives of PeKa B40 is screening of mental health.	6 (2.0)	20 (6.8)	78 (26.4)	117 (39.5)	75 (25.3)
4	ProtectHealth Corporation that manages the PeKa B40 scheme is not a Third Party Administrator.	20 (6.8)	45 (15.2)	134 (45.3)	57 (19.3)	40 (13.5)
5	The PeKa B40 scheme covers those from the minimum age of 40 years old .	25 (8.4)	19 (6.4)	65 (22.0)	98 (33.1)	89 (30.1)
