# Supplementary material for: Gatekeepers in the health financing scheme: Assessment of knowledge, attitude, practices, and participation of Malaysian private general practitioners in the PeKa B40 scheme
Source: PLoS One. 2023 Oct 17;18(10):e0292516. doi: 10.1371/journal.pone.0292516 (PMC10581488; doi:10.1371/journal.pone.0292516)
Supplement: S4 Table — This table contains the responses according to the 5-point Likert scale, on the various sources of information for each category ie. health financing schemes, gatekeeper role and PeKa B40. (PDF) [file pone.0292516.s004.pdf]

S4 Table Practice issues in PeKa B40 (N=296) This table list the responses according to the 5-point Likert scale on various aspects of the consultation process in PeKa B40, including history, physical examination and investigation.

No	Item	n (%)				
		**Strongly disagree**	**Disagree**	**Neutral**	**Agree**	**Strongly agree**
1	History A: Symptoms of the chronic disease	0	1 (0.3)	16 (5.4)	107 (36.1)	172 (58.1)
2	History B: Past medical history	0	1 (0.3)	13 (4.4)	104 (35.1)	178 (60.1)
3	History C: Family history of specific medical conditions	0	1 (0.3)	15 (5.1)	103 (34.8)	177 (59.8)
4	History D: Behavioral risk factors	1 (0.3)	0	20 (6.8)	108 (36.5)	167 (56.4)
5	History E: Mental health assessment	2 (0.7)	2 (0.7)	30 (10.1)	105 (35.5)	157 (53.0)
6	Physical examination A: Weight / Height / Body mass index	0	0	10 (3.4)	95 (32.1)	191 (64.5)
7	Physical examination B: Vital signs: Blood pressure, temperature, pulse rate	0	0	10 (3.4)	88 (29.7)	198 (66.9)
8	Physical examination C: General physical examination	0	0	12 (4.1)	91 (30.7)	193 (65.2)
9	Physical examination D: Specific systemic examination	1 (0.3)	0	25 (8.4)	101 (34.1)	169 (57.1)
10	Physical examination E: Clinical breast examination (for females)	1 (0.3)	5 (1.7)	45 (15.2)	104 (35.1)	141 (47.6)
11	Physical examination F: Digital rectal examination	10 (3.4)	21 (7.1)	121 (40.9)	73 (24.7)	71 (24.0)
12	Investigation A: Urine biochemistry	0	0	26 (8.8)	109 (36.8)	161 (54.4)
13	Investigation B: Full blood count	0	2 (0.7)	27 (9.1)	111 (37.5)	156 (52.7)
14	Investigation C: Renal profile	0	0	27 (9.1)	105 (35.5)	164 (55.4)
15	Investigation D: HbA1 C	0	5 (1.7)	30 (10.1)	107 (36.1)	154 (52.0)
16	Investigation E: Lipid profile	0	0	25 (8.4)	100 (33.8)	171 (57.8)
