# Supplementary material for: Gatekeepers in the health financing scheme: Assessment of knowledge, attitude, practices, and participation of Malaysian private general practitioners in the PeKa B40 scheme
Source: PLoS One. 2023 Oct 17;18(10):e0292516. doi: 10.1371/journal.pone.0292516 (PMC10581488; doi:10.1371/journal.pone.0292516)
Supplement: S5 Table — This table assesses the responses according to the 5-point Likert scale in reference to the attitude towards remuneration, tax-based finance, social health insurance and private insurance. (PDF) [file pone.0292516.s005.pdf]

S5 Table Attitude towards health financing scheme This table assesses the responses according to the 5-point Likert scale in reference to the attitude towards remuneration, tax-based finance, social health insurance and private insurance.

No	Item	n (%)				
		Strongly disagree	Disagree	Neutral	Agree	Strongly agree
1	Remuneration for Malaysian private GPs should be based on Capitation on consultation	11 (3.7)	21 (7.1)	53 (17.9)	119 (40.2)	92 (31.1)
2	Remuneration for Malaysian private GPs should be based on Fee for Service for Procedures & minor surgeries.	3 (1.0)	4 (1.4)	29 (9.8)	125 (42.2)	135 (45.6)
3	Remuneration for Malaysian private GPs should include Health promotion and Disease	0	2 (0.7)	36 (12.2)	113 (38.2)	145 (49.0)
4	Remuneration for Malaysian GPs should include medication dispensing	145 (49.0)	3 (1.0)	24 (8.1)	100 (33.8)	164 (55.4)
5	Tax-based finance is the best method for Malaysia	7 (2.4)	17 (5.7)	168 (56.8)	66 (22.3)	38 (12.8)
6	Tax-based finance in Malaysia offers government servants better benefits than private workers	9 (3.0)	18 (6.1)	140 (47.3)	78 (26.4)	51 (17.2)
7	In tax-based finance, income-based tax is preferred to consumption tax because it allows collection from a larger income base.	10 (3.4)	11 (3.7)	143 (48.3)	96 (32.4)	36 (12.2)
8	In tax-based finance , the Goods and Services tax should be implemented as an additional revenue source in Malaysia.	22 (7.4)	27 (9.1)	130 (43.9)	70 (23.6)	47 (15.9)
9	Social health insurance (SHI) should be introduced in Malaysia to replace the tax-based finance system	11 (3.7)	17 (5.7)	143 (48.3)	75 (25.3)	50 (16.9)
10	SHI should be introduced in Malaysia in combination with the current tax-based system	9 (3.0)	10 (3.4)	122 (41.2)	105 (35.5)	50 (16.9)
11	If SHI is introduced in Malaysia, the poor should be covered by the government	4 (1.4)	5 (1.7)	66 (22.3)	108 (36.5)	113 (38.2)
12	In SHI, a single payor involving only one entity as the paymaster is preferred over multi-payors which involve several paymasters.	5 (1.7)	15 (5.1)	150 (50.7)	75 (25.3)	75 (25.3)
13	Multiple benefit packages with differing coverage is preferred over a single package in SHI	5 (1.7)	11 (3.7)	109 (36.8)	112 (37.8)	59 (19.9)

No	Item	n (%)				
		Strongly disagree	Disagree	Neutral	Agree	Strongly agree
14	Private insurance in Malaysia should not substitute the current tax-based system in Malaysia	7 (2.4)	12 (4.1)	116 (39.2)	87 (29.4)	74 (25.0)
15	Private insurance is preferred to play only a supplementary role	4 (1.4)	20 (6.8)	93 (31.4)	110 (37.2)	69 (23.3)
