# Supplementary material for: Gatekeepers in the health financing scheme: Assessment of knowledge, attitude, practices, and participation of Malaysian private general practitioners in the PeKa B40 scheme
Source: PLoS One. 2023 Oct 17;18(10):e0292516. doi: 10.1371/journal.pone.0292516 (PMC10581488; doi:10.1371/journal.pone.0292516)
Supplement: S6 Table — This table list the responses according to the 5-point Likert scale, on the attitude towards various aspects of the gatekeeper roles. (PDF) [file pone.0292516.s006.pdf]

S6 Table Attitude towards gatekeeper roles (N=296) This table list the responses according to the 5-point Likert scale , on the attitude towards various aspects of the gatekeeper roles.

No	Item	n (%)				
		Strongly disagree	Disagree	Neutral	Agree	Strongly agree
1	In Malaysia, GPs should assume the full gatekeeper role whereby all patients must first be triaged by the GPs	0	17 (5.7)	47 (15.9)	111 (37.5)	121 (40.9)
2	The gatekeeper system will benefit the practice of private Malaysian GPs	0	5 (1.7)	35 (11.8)	125 (42.2)	131 (44.3)
3	Private GPs in Malaysia must be vocationally-trained family physicians for effective gatekeeper roles.	13 (4.4)	29 (9.8)	75 (25.3)	95 (32.1)	84 (28.4)
4	The gatekeeper role for Malaysian GPs is only effective if the National Health Finance system is implemented.	10 (3.4)	13 (4.4)	103 (34.8)	92 (31.1)	78 (26.4)
