# Supplementary material for: Gatekeepers in the health financing scheme: Assessment of knowledge, attitude, practices, and participation of Malaysian private general practitioners in the PeKa B40 scheme
Source: PLoS One. 2023 Oct 17;18(10):e0292516. doi: 10.1371/journal.pone.0292516 (PMC10581488; doi:10.1371/journal.pone.0292516)
Supplement: S7 Table — This table list the various responses according to the 5-point Likert scale on the attitude towards various aspects of the PeKa B40 scheme. (PDF) [file pone.0292516.s007.pdf]

S7 Table Attitude towards PeKa B40 (N=296) This table list the various responses according to the 5-point Likert scale on the attitude towards various aspects of the PeKa B40 scheme.

No	Item	n (%)				
		Strongly disagree	Disagree	Neutral	Agree	Strongly agree
1	The current limited screening role of the Malaysian private GPs in PeKa B40 is acceptable.	25 (8.4)	53 (17.9)	132 (44.6)	65 (22.0)	21 (7.1)
2	The current remuneration of RM60 for private GPs in PeKa B40 commensurate with the amount of work done.	45 (15.2)	72 (24.3)	111 (37.5)	53 (17.9)	15 (5.1)
3	The registration process for private GPs into the PeKa B40 provider scheme is not complicated.	16 (5.4)	36 (12.2)	140 (47.3)	74 (25.0)	30 (10.1)
4	Participating private GPs in PeKa B40 should be remunerated to fully manage the detected cases with the appropriate medication.	2 (0.7)	6 (2.0)	70 (23.6)	123 (41.6)	95 (32.1)
5	The role of private GPs in PeKa B40 should be extended to health promotion activities such as exercise.	3 (1.0)	6 (2.0)	60 (20.3)	128 (43.2)	99 (33.4)
6	The role of private GPs in PeKa B40 should also include all levels of Disease Prevention instead of just performing screening tests.	2 (0.7)	1 (0.3)	43 (14.5)	125 (42.2)	125 (42.2)
7	Private GPs engaging in Health promotion and Disease prevention activities in PeKa B40 should be remunerated accordingly.	0	0	42 (14.2)	126 (42.6)	128 (43.2)
8	Private GPs in PeKa B40 should be incorporated in multidisciplinary long term care initiatives with public healthcare facilities	1 (0.3)	1 (0.3)	47 (15.9)	128 (43.2)	119 (40.2)
