# Supplementary material for: Gatekeepers in the health financing scheme: Assessment of knowledge, attitude, practices, and participation of Malaysian private general practitioners in the PeKa B40 scheme
Source: PLoS One. 2023 Oct 17;18(10):e0292516. doi: 10.1371/journal.pone.0292516 (PMC10581488; doi:10.1371/journal.pone.0292516)
Supplement: S8 Table — This table list the responses according to the 5-point Likert scale on various aspects of the consultation process in PeKa B40, including history, physical examination and investigation. (PDF) [file pone.0292516.s008.pdf]

S8 Table Acceptance and level of participation to the National Health Finance Scheme. (NHFS) (N=296) This table list the responses according to the 5-point Likert scale on the various issues related to the NHFS. This was to assess acceptance and level of participation in the NHFS.

No	Item	n (%)				
		Strongly disagree	Disagree	Neutral	Agree	Strongly agree
1	A National Health Finance System (NHFS) SHOULD be implemented in Malaysia.	6 (2.0)	12 (4.1)	88 (29.7)	95 (32.1)	95 (32.1)
2	We NEED to implement a National Health Finance System (NHFS) URGENTLY.	13 (4.4)	22 (7.4)	113 (38.2)	78 (26.4)	70 (23.6)
3	I am committed to assume the Gatekeeper role in the NHFS.	4 (1.4)	2 (0.7)	79 (26.7)	108 (36.5)	103 (34.8)
4	I am fully aware of the components of NHFS	9 (3.0)	40 (13.5)	132 (44.6)	67 (22.6)	48 (16.2)
5	I am willing to advance my training in areas related to NHFS.	3 (1.0)	8 (2.7)	94 (31.8)	109 (36.8)	82 (27.7)
6	The PEKA B40 programme has the potential to develop into the NHFS	10 (3.4)	10 (3.4)	108 (36.5)	101 (34.1)	67 (22.6)
