# Supplementary material for: Gatekeepers in the health financing scheme: Assessment of knowledge, attitude, practices, and participation of Malaysian private general practitioners in the PeKa B40 scheme
Source: PLoS One. 2023 Oct 17;18(10):e0292516. doi: 10.1371/journal.pone.0292516 (PMC10581488; doi:10.1371/journal.pone.0292516)
Supplement: S9 Table — This table list the responses according to the 5-point Likert scale on the various issues related to the NHFS. This was to assess acceptance and level of participation in the NHFS. (PDF) [file pone.0292516.s009.pdf]

S9 Table Sources of information (N=296) This table contains the responses according to the 5-point Likert scale, on the various sources of information for each category ie health financing schemes , gatekeeper role and PeKa B40.

No	Item	n (%)				
		**Strongly disagree**	**Disagree**	**Neutral**	**Agree**	**Strongly agree**
	**Health financing scheme**					
1	Internet	3 (1.0)	1 (0.3)	33 (11.1)	119 (40.2)	140 (47.3)
2	Mass media	2 (0.7)	9 (3.0)	75 (25.3)	114 (38.5)	96 (32.4)
3	Social media	6 (2.0)	16 (5.4)	62 (20.9)	115 (38.9)	97 (32.8)
4	Courses	2 (0.7)	7 (2.4)	67 (22.6)	103 (34.8)	117 (39.5)
5	Societies	3 (1.0)	14 (4.7)	85 (28.7)	116 (39.2)	78 (26.4)
6	Peers	0	8 (2.7)	82 (27.7)	129 (43.6)	77 (26.0)
7	Journals	9 (3.0)	16 (5.4)	76 (25.7)	113 (38.2)	82 (27.7)
	**Gatekeeper roles**					
1	Internet	1 (0.3)	0	38 (12.8)	124 (41.9)	133 (44.9)
2	Mass media	3 (1.0)	4 (1.4)	84 (28.4)	112 (37.8)	93 (31.4)
3	Social media	9 (3.0)	9 (3.0)	74 (25.0)	112 (37.8)	89 (30.1)
4	Courses	4 (1.4)	6 (2.0)	70 (23.6)	112 (37.8)	104 (35.1)
5	Societies	5 (1.7)	11 (3.7)	94 (31.8)	111 (37.5)	75 (25.3)
6	Peers	2 (0.7)	6 (2.0)	81 (27.4)	125 (42.2)	82 (27.7)
7	Journals	6 (2.0)	13 (4.4)	83 (28.0)	102 (34.5)	92 (31.1)
	**PeKa B40**					
1	Internet	1 (0.3)	5 (1.7)	49 (16.6)	110 (37.2)	131 (44.3)
2	Mass media	2 (0.7)	4 (1.4)	71 (24.0)	121 (40.9)	98 (33.1)
3	Social media	7 (2.4)	13 (4.4)	74 (25.0)	105 (35.5)	97 (32.8)
4	Courses	8 (2.7)	16 (5.4)	103 (34.8)	90 (30.4)	79 (26.7)
5	Societies	4 (1.4)	14 (4.7)	97 (32.8)	102 (34.5)	79 (26.7)
6	Peers	2 (0.7)	9 (3.0)	87 (29.4)	114 (38.5)	84 (28.4)
7	Journals	17 (5.7)	20 (6.8)	115 (38.9)	82 (27.7)	62 (20.9)
