# Supplementary material for: Gatekeepers in the health financing scheme: Assessment of knowledge, attitude, practices, and participation of Malaysian private general practitioners in the PeKa B40 scheme
Source: PLoS One. 2023 Oct 17;18(10):e0292516. doi: 10.1371/journal.pone.0292516 (PMC10581488; doi:10.1371/journal.pone.0292516)
Supplement: S1 File — (PDF) [file pone.0292516.s010.pdf]

S10 Ethics statement

The information sheet and consent forms were attached as initial pages of the questionnaires. The respondents would indicate their written consent on the attached forms and subsequently respond to the survey questionnaires. Upon completion, the information sheet and written consent forms were submitted together with the responses. The consent of each participant was ensured that it was duly filled before the responses were accepted.
