# Supplementary material for: Gatekeepers in the health financing scheme: Assessment of knowledge, attitude, practices, and participation of Malaysian private general practitioners in the PeKa B40 scheme
Source: PLoS One. 2023 Oct 17;18(10):e0292516. doi: 10.1371/journal.pone.0292516 (PMC10581488; doi:10.1371/journal.pone.0292516)
Supplement: S2 File — (PDF) [file pone.0292516.s011.pdf]

S11 Respondent Information Sheet.

Respondent Information Sheet

Study Title: Healthcare Financing in Malaysia: Participation in PEKA B40 Scheme and Knowledge, Attitude and Practices of Gatekeeper Role among Private General Practitioners

Introduction:

Private General Practitioners (GPs) are an important contributors to the primary care service in Malaysia. There are an estimated more than 6,000 private GPs in the country.

Of late, there has been talk by the Ministry of Health to reform the healthcare system with emphasis on primary care and a move towards introducing a National Health Finance scheme. Currently, Malaysia is supported by a tax-based finance scheme in the public sector in which the health service is accessible to all citizens, as well as private insurances which cater for selected groups of people. Additionally, there has also been suggestions of introducing Social Health insurance into the current system as well.

Regardless of the type of National Health insurance, the role of primary care doctors including GPs, will need to be defined specifically to ensure that the system is effective.

As an initial step, the government had introduced PEKA B40 to cater for the the B40 group; a screening program involving GPs for early detection of undiagnosed non-communicable diseases amongst the population. This program is a pilot initiative in public- private partnership at the primary care level. This study aims to assess the preparedness of the private GPs as gatekeepers of a national health insurance scheme.

What would this involve?

This is a mixed-methods study which will involve the participation of private GPs in Malaysia at two levels:

1. Survey questionnaires will be mailed to participating Malaysian private GPs to assess their **knowledge, attitude and practice** on health finance systems in general, **gatekeeper role** in health finance systems and **PEKA B40.**
2. Focus groups discussions involving selected GPs divided who are PEKA B40 providers and non-PEKA B40 providers.

The benefits

This study will give an overall assessment of the levels of readiness on these matters and provide feedback for remedial efforts in deficient areas. Hence private GPs will benefit from this assessment and policy makers will be able to execute proposed reforms.

The risks

There are no anticipated risks as there are no interventions involved.

Confidentiality

All the information that we collect from this survey will be kept confidential and will only be reported as group data with no identifying information. All data will be kept in a secure location and only those directly involved with the survey will have access to them.

Do I have to take part?

The participation in this study is voluntary. Any respondent may also choose to opt out during any phase of the study without any reasons.

If I have any questions, whom can I ask at any time of the study?

You may contact the following:

Associate Professor Dr Aznida Firzah Abdul Aziz

Department of Family Medicine, Faculty of Medicine, UKM

Email: draznida@ppukm.ukm.edu.my

Tel: 03 9145 6117 (O), 011 5970 7386 (mobile)

Dr Mohammad Husni Jamal (P90483)

Phd Candidate, Dept. Community Health, Faculty of Medicine, UKM

Email: drmhusnij@gmail.com Mobile: +60192822255
