# Supplementary material for: Gatekeepers in the health financing scheme: Assessment of knowledge, attitude, practices, and participation of Malaysian private general practitioners in the PeKa B40 scheme
Source: PLoS One. 2023 Oct 17;18(10):e0292516. doi: 10.1371/journal.pone.0292516 (PMC10581488; doi:10.1371/journal.pone.0292516)
Supplement: S3 File — (PDF) [file pone.0292516.s012.pdf]

S12 Informed Consent Form.

Informed consent form

Research Title: Healthcare Financing in Malaysia: Participation in PEKA B40 Scheme and Knowledge, Attitude and Practices of Gatekeeper Role among Private General Practitioners

Researcher's Name: Dr Mohammad Husni Jamal (P90483)

I, ……………………………………, IC No : ………………….....

- have read the information in the Respondent Information Sheet including information regarding the risk in this study
- have been given time to think about it and all of my questions have been answered to my satisfaction.
- understand that I may freely choose to withdraw from this study at anytime without reason and without repercussion
- understand that my anonymity will be ensured in the write-up.

I voluntarily agree to be part of this research study, to follow the study procedures, and to provide necessary information to the doctor, nurses, or other staff members, as requested.

……………………………… ………………….

(Signature) (Date)
