# Supplementary material for: Gatekeepers in the health financing scheme: Assessment of knowledge, attitude, practices, and participation of Malaysian private general practitioners in the PeKa B40 scheme
Source: PLoS One. 2023 Oct 17;18(10):e0292516. doi: 10.1371/journal.pone.0292516 (PMC10581488; doi:10.1371/journal.pone.0292516)
Supplement: Attachment — Submitted filename: Plos One responses to Reviewers Final 300723.docx [file pone.0292516.s013.docx]

**PONE-D-23-12708: Gatekeepers in Health Financing Scheme: Assessment of Knowledge, Attitude, Practices and Participation of Malaysian private GPs in PeKa B40 Scheme**

| **Experts** | **Comments** | **Changes in the manuscript** | **Authors explanation** |
| --- | --- | --- | --- |
| **Journal requirements** | 1. Please ensure that your manuscript meets PLOS ONE's style requirements, including those for file naming. The PLOS ONE style templates can be found at  https://journals.plos.org/plosone/s/file?id=wjVg/PLOSOne_formatting_sample_main_body.pdf and  <https://journals.plos.org/plosone/s/file?id=ba62/PLOSOne_formatting_sample_title_authors_affiliations.pdf>  2. Please provide additional details regarding participant consent. In the ethics statement in the Methods and online submission information, please ensure that you have specified (1) whether consent was informed and (2) what type you obtained (for instance, written or verbal, and if verbal, how it was documented and witnessed). If your study included minors, state whether you obtained consent from parents or guardians. If the need for consent was waived by the ethics committee, please include this information.  If you are reporting a retrospective study of medical records or archived samples, please ensure that you have discussed whether all data were fully anonymized before you accessed them and/or whether the IRB or ethics committee waived the requirement for informed consent. If patients provided informed written consent to have data from their medical records used in research, please include this information  Once you have amended this/these statement(s) in the Methods section of the manuscript, please add the same text to the “Ethics Statement” field of the submission form (via “Edit Submission”).  For additional information about PLOS ONE ethical requirements for human subjects research, please refer to <http://journals.plos.org/plosone/s/submission-guidelines#loc-human-subjects-research>.  3. Thank you for stating the following financial disclosure:  "Yes. The study was funded by the Faculty of Medicine, Universiti Kebangsaan Malaysia FF-2020-403 under supervisor's name Azimatun Noor Aizuddin for PhD  student's research."  Please state what role the funders took in the study. If the funders had no role, please state: "The funders had no role in study design, data collection and analysis, decision to publish, or preparation of the manuscript."  If this statement is not correct you must amend it as needed.  Please include this amended Role of Funder statement in your cover letter; we will change the online submission form on your behalf.  4. In your Data Availability statement, you have not specified where the minimal data set underlying the results described in your manuscript can be found. PLOS defines a study's minimal data set as the underlying data used to reach the conclusions drawn in the manuscript and any additional data required to replicate the reported study findings in their entirety. All PLOS journals require that the minimal data set be made fully available. For more information about our data policy, please see http://journals.plos.org/plosone/s/data-availability.  "Upon re-submitting your revised manuscript, please upload your study’s minimal underlying data set as either Supporting Information files or to a stable, public repository and include the relevant URLs, DOIs, or accession numbers within your revised cover letter. For a list of acceptable repositories, please see http://journals.plos.org/plosone/s/data-availability#loc-recommended-repositories. Any potentially identifying patient information must be fully anonymized.  Important: If there are ethical or legal restrictions to sharing your data publicly, please explain these restrictions in detail. Please see our guidelines for more information on what we consider unacceptable restrictions to publicly sharing data: http://journals.plos.org/plosone/s/data-availability#loc-unacceptable-data-access-restrictions. Note that it is not acceptable for the authors to be the sole named individuals responsible for ensuring data access.  We will update your Data Availability statement to reflect the information you provide in your cover letter.  5. Please amend either the title on the online submission form (via Edit Submission) or the title in the manuscript so that they are identical.  6. Your ethics statement should only appear in the Methods section of your manuscript. If your ethics statement is written in any section besides the Methods, please move it to the Methods section and delete it from any other section. Please ensure that your ethics statement is included in your manuscript, as the ethics statement entered into the online submission form will not be published alongside your manuscript.  7. Please review your reference list to ensure that it is complete and correct. If you have cited papers that have been retracted, please include the rationale for doing so in the manuscript text, or remove these references and replace them with relevant current references. Any changes to the reference list should be mentioned in the rebuttal letter that accompanies your revised manuscript. If you need to cite a retracted article, indicate the article’s retracted status in the References list and also include a citation and full reference for the retraction notice. | All formatting done accordingly.  .  The Ethics statement, Respondent Information sheet and Informed Consent were added under Supplementary files S10, S11 and S12 respectively.  The Data availability statement is attached as Supporting file S13.  The title is as follows:  **Gatekeepers in the Health Financing Scheme: Assessment of Knowledge, Attitude, Practices, and Participation of Malaysian Private General Practitioners in the PeKa B40 Scheme**  **Gatekeepers, KAP and GP participation in Health Financing Scheme**.  The Ethics statement is relocated to the Methods section in the manuscript.  2.5 Ethical Approval  Ethical approval was awarded by the UKM Ethics Committee letter dated August 28, 2020 (ref no UKM PPI/111/8/JEP-2020-539).  Ethics statement:  The information sheet and consent forms were attached as initial pages of the questionnaires. The respondents would indicate their consent on the attached forms and subsequently respond to the survey questionnaires. Upon completion, the information sheet and consent forms were submitted together with the responses. The consent of each participant was ensured that it was duly filled before the responses were accepted (documents as attached in Supporting Information S10, S11 and S12).  **These 3 earlier references had been deleted from the original manuscript.**    5.Malaysia National Health Accounts. Ministry of Health.  Health Expenditure Report 1997-2017. 2019. Ch 8: Pg 75. [ Cited 2021 December 18] Available from:  https://www.moh.gov.my/moh/resources/Penerbitan/Penerbitan%20Utama/MNHA/Laporan_MNHA_Health_Expenditure_Report_1997-2017_03122019.  6.Anon. Malaysia’s Health Spending Estimated To Hit 5% GDP in 2021. CodeBlue. 2021. September 21. [Cited 2022 July 14] Available from:  https://codeblue.galencentre.org/2021/09/21/malaysias-health-spending-estimated-to-hit-5-gdp-in-2021/  7.Jaafar, S., Noh, K.M., Muttalib, K.A., Othman, N. & Heal, J. Malaysia. Malaysia Health System Review, Health Systems in Transition. 2013. Vol. 3 No. 1 pg. 39 [ Cited 2020 October 21]. Available from:  https://apps.who.int/iris/bitstream/handle/10665/206911/9789290615842_eng.pdf?sequence=1&isAllowed=y  **New references have been added and numbered accordingly.**  **They are listed as follows:**  5. Ministry of Health Malaysia. Health White Paper for Malaysia: Strengthening people’s health, future-proofing the nation’s health system. 2023.Ministry of Health for the Government of Malaysia. Available from: kertasputih@moh.gov.my  6. Chua, H.T., Cheah, J.C.H. Financing Universal Coverage in Malaysia: a case study. BMC Public Health 12 (Suppl 1), S7 (2012).  8. Croke, K., Mariana Binti Mohd Yusoff, Zalilah Abdullah, Ainul Nadziha, Mohd Hanafiah, Khairiah Mokhtaruddin et al.The political economy of health financing reform in Malaysia. Health Policy and Planning. 2019. December 34(10):732-739  14. Ong SM, Lim MT, Fah Tong S, Kamaliah MN, Groenewegen P, Sivasampu S. Comparative performance of public and private primary care service delivery in Malaysia: An analysis of findings from QUALICOPC. PLoS ONE. 2022. 17(10): e0276480. https://doi.org/10.1371/journal. pone.0276480)  15. Makmor Tumin, Ganeshwaran Kana, Aimi Ahmad Zaki. Financing and Provision of Primary Health Care in Malaysia. Journal of Administrative Science.2016. Vol.13, Issue 2, Available online at http:jas.uitm.edu.my)  17. Willems, D. Balancing rationalities: gatekeeping in health care. J Med Ethics. 2001 Feb;27(1):25-9.)  18. Ang, K.T. Ho, B.K. Mimi, O. Salmah, N. Salmiah, M.S. Noridah, M.S.. Factors influencing the role of primary care providers as gatekeepers in the Malaysian public healthcare system. Malaysian Fam Physician. 2014. 9(3): 2–11).  19. Kassai R, van Weel C, Flegg K, Tong SF, Han TM, Noknoy S, et al. Priorities for primary health care policy implementation: recommendations from the combined experience of six countries in the Asia-Pacific. Aust J Prim Health. 2020 Oct;26(5):351-357. doi: 10.1071/PY19194. PMID: 32746962  20. Kenny, K., Omar, Z., Kanavathi, E. S., & Madhavan, P.. A study on the health economics of General Practitioners in the health care services in Malaysia: Trends, Challenges and Moving Forward. International Journal of Public Health Research. 2017. 7(1), 765–774. Available from: https://spaj.ukm.my/ijphr/index.php/ijphr/article/view/72)  21. healthdirect .What is Medicare? [cited 2023 July 11] Available from: https://www.healthdirect.gov.au/what-is-medicare  22. Jolyon Attwooll. Government flags shift in fee-for-service model.RACGP. 2023. Jan 23. [cited 2023 July 11]. Available from: https://www1.racgp.org.au/newsgp/professional/government-flags-shift-in-fee-for-service-model  23. Thaworn Sakunphanit.Thailand’s Universal Health Coverage- Regional Seminar “Rethinking Social Protection in a Changing Arab Region” Amman, Jordan 13 -15 May 2014. Available from:  https://www.ilo.org/wcmsp5/groups/public/---arabstates/---ro-beirut/documents/genericdocument/wcms_246594.pdf  24. Mubarak N, Raja SA, Khan AS, Kanwal S, Saif-Ur-Rehman N, Aziz MM et al.  A Conceptual Framework of the Way Forward to a Community Pharmacist-General Practitioner Collaborative Medication Therapy Management Model for Chronic Diseases in Malaysian Primary Care: A Qualitative Study. Risk Manag Healthc Policy. 2021 Apr 19;14:1615-1627. doi: 10.2147/RMHP.S296113. PMID: 33907479; PMCID: PMC8064717.  28. Savedoff, W. Tax-Based Financing for Health Systems: Options and Experiences. Discussion Paper No.4. 2004.World Health Organisation)  29. Abrejo, F.G. & Shaikh, B.T.. Social health insurance: can we ever make a case for Pakistan? J Pak Med Assoc. 2008.May;58(5):267-70  30. Duggal, R.. Private health insurance and access to healthcare. Indian J Med Ethics. 2011. 8(1):28-30.  31. Willems, D.L. Balancing rationalities: gatekeeping in health care. J Med Ethics. 2001. Feb;27(1):25-9.  32. PeKa B40. https://protecthealth.com.my/peka-b40.html  33. Mathauer, I. Schmidt, J.O. & Wenyaa, M. Extending social health insurance to the informal sector in Kenya. An assessment of factors affecting demand. Int J Health Plann Manage. 2008. Jan-Mar;23(1):51-68. | We have formatted the revised manuscript accordingly.  Informed consent was obtained via written documentation, which included the information on the nature of the study and a section for the respondent to sign his/her consent to participate.  No minors were included.  The need for informed consent was not waived by the Ethics committee.  The above disclosures were mentioned in both the Methods and online submission accordingly.  Done as advised.  Done as advised.  The 3 earlier articles were replaced by more relevant current references.  New articles were added in response to the 2 Reviewers comments and listed accordingly.  All the new references were in response to the suggestions of both Reviewers 1 and 2. |
| **Additional editor comments** | I have now received the feedback from the reviewers, and after careful consideration, I have decided to revise the manuscript based on their comments.  Firstly, I would like to commend you on the valuable research you have conducted on the knowledge, attitude, practices, and participation of Malaysian private General Practitioners (GPs) in the PeKa B40 Scheme. The topic is timely and relevant, addressing an important aspect of health financing in Malaysia. The reviewers appreciated the significance of your study and its potential contribution to the existing literature.  However, there are several points that require attention and revision before the manuscript can be considered suitable for publication. |  | - |
| **Reviewer #1** | The literature cited does not depict an exhaustive coverage of the topic in hand. You need to bring more Malaysian studies and discuss in the discussion section. | These Malaysian studies were added.  5. Ministry of Health Malaysia. Health White Paper for Malaysia: Strengthening people’s health, future-proofing the nation’s health system. 2023.Ministry of Health for the Government of Malaysia. Available from: kertasputih@moh.gov.my  6. Chua, H.T., Cheah, J.C.H. Financing Universal Coverage in Malaysia: a case study. BMC Public Health 12 (Suppl 1), S7 (2012).  8. Croke, K., Mariana Binti Mohd Yusoff, Zalilah Abdullah, Ainul Nadziha, Mohd Hanafiah, Khairiah Mokhtaruddin et al.The political economy of health financing reform in Malaysia. Health Policy and Planning. 2019. December 34(10):732-739  14. Ong SM, Lim MT, Fah Tong S, Kamaliah MN, Groenewegen P, Sivasampu S. Comparative performance of public and private primary care service delivery in Malaysia: An analysis of findings from QUALICOPC. PLoS ONE. 2022. 17(10): e0276480. https://doi.org/10.1371/journal. pone.0276480)  15. Makmor Tumin, Ganeshwaran Kana, Aimi Ahmad Zaki. Financing and Provision of Primary Health Care in Malaysia. Journal of Administrative Science.2016. Vol.13, Issue 2, Available online at http:jas.uitm.edu.my)  17. Willems, D. Balancing rationalities: gatekeeping in health care. J Med Ethics. 2001 Feb;27(1):25-9.)  18. Ang, K.T. Ho, B.K. Mimi, O. Salmah, N. Salmiah, M.S. Noridah, M.S.. Factors influencing the role of primary care providers as gatekeepers in the Malaysian public healthcare system. Malaysian Fam Physician. 2014. 9(3): 2–11).  19. Kassai R, van Weel C, Flegg K, Tong SF, Han TM, Noknoy S, et al. Priorities for primary health care policy implementation: recommendations from the combined experience of six countries in the Asia-Pacific. Aust J Prim Health. 2020 Oct;26(5):351-357. doi: 10.1071/PY19194. PMID: 32746962  20. Kenny, K., Omar, Z., Kanavathi, E. S., & Madhavan, P.. A study on the health economics of General Practitioners in the health care services in Malaysia: Trends, Challenges and Moving Forward. International Journal of Public Health Research. 2017. 7(1), 765–774. Available from: https://spaj.ukm.my/ijphr/index.php/ijphr/article/view/72)  21. healthdirect .What is Medicare? [cited 2023 July 11] Available from: https://www.healthdirect.gov.au/what-is-medicare  22. Jolyon Attwooll. Government flags shift in fee-for-service model.RACGP. 2023. Jan 23. [cited 2023 July 11]. Available from: https://www1.racgp.org.au/newsgp/professional/government-flags-shift-in-fee-for-service-model  23. Thaworn Sakunphanit.Thailand’s Universal Health Coverage- Regional Seminar “Rethinking Social Protection in a Changing Arab Region” Amman, Jordan 13 -15 May 2014. Available from:  https://www.ilo.org/wcmsp5/groups/public/---arabstates/---ro-beirut/documents/genericdocument/wcms_246594.pdf  24. Mubarak N, Raja SA, Khan AS, Kanwal S, Saif-Ur-Rehman N, Aziz MM et al.  A Conceptual Framework of the Way Forward to a Community Pharmacist-General Practitioner Collaborative Medication Therapy Management Model for Chronic Diseases in Malaysian Primary Care: A Qualitative Study. Risk Manag Healthc Policy. 2021 Apr 19;14:1615-1627. doi: 10.2147/RMHP.S296113. PMID: 33907479; PMCID: PMC8064717.  28. Savedoff, W. Tax-Based Financing for Health Systems: Options and Experiences. Discussion Paper No.4. 2004.World Health Organisation)  29. Abrejo, F.G. & Shaikh, B.T.. Social health insurance: can we ever make a case for Pakistan? J Pak Med Assoc. 2008.May;58(5):267-70  30. Duggal, R.. Private health insurance and access to healthcare. Indian J Med Ethics. 2011. 8(1):28-30.  31. Willems, D.L. Balancing rationalities: gatekeeping in health care. J Med Ethics. 2001. Feb;27(1):25-9.  32. PeKaB40. https://protecthealth.com.my/peka-b40.html  33. Mathauer, I. Schmidt, J.O. & Wenyaa, M. Extending social health insurance to the informal sector in Kenya. An assessment of factors affecting demand. Int J Health Plann Manage. 2008. Jan-Mar;23(1):51-68. | All these articles had been added to the revised manuscript.  Other new articles were also added in response to Reviewer 2.  We had attempted to incorporate both Reviewers 1 and 2 comments in the Discussion section. |
|  | Sample Selection: How was the stratified random sampling technique implemented to ensure the sample of GPs represents the population adequately? Were there any limitations or biases in the sampling method that could impact the generalizability of the findings? | The initial response from the federal societies such as the Malaysian Medical Association (MMA) and FPMPAM was poor. In order to obtain a bigger number of responses for sampling, we approached each state via different networks such as the state-level MMAs and the other formal GP groups. Sources were also obtained from other various informal GP connections. Hence, we had to adopt different means from state to state but ultimately we managed to obtain the required number for the stratified sampling. The stratified sampling selected 13 participants each from urban/rural in which we managed to select a total of 362 samples. However, we had 66 non-responders despite several reminders. Whilst we should have attempted to recruit more participants, the difficulty that was faced was the survey was carried out over the period of the Covid lockdown, which was unpredicted for but we had no choice but to continue. It was very difficult to get more GP responders as the mood amongst GPs was very passive. | These points were added in the section on 2.1 on Population sampling. |
|  | Questionnaire Validity and Reliability: How were the self-designed questionnaires developed, and were they validated and tested for reliability? Can the researchers provide details on the measures taken to ensure the accuracy and consistency of the questionnaire responses? |  | This was stated in Section 2.2 of the manuscript. |
|  | Response Rate and Non-Response Bias: Although the response rate of 81.7% is reasonable, it is important to consider potential non-response bias. Were there any significant differences between respondents and non-respondents that could introduce bias into the findings? | Nonresponse bias occurs when survey participants are unwilling or unable to respond to a survey question or an entire survey. Whilst we should have attempted to recruit more participants, the difficulty that was faced was the survey was carried out over the period of the Covid lockdown, which was unpredicted for but we had no choice but to continue. It was very difficult to get more GP responders as the mood amongst GPs was very passive. We had ruled out other common causes of non-response bias, including:  a. Poor survey design  We conducted a pilot test involving 30 GPs. We did not encounter any specific complaints on the survey questionnaires including the time duration taken to complete the survey. We also avoided structuring double-barreled questionnaires  b..Failed delivery  Non-responders were monitored and communicated with, and some had initially experienced such surveys being sent in their spam boxes but were retrieved.  c. Set expectations  The survey information and consent forms were attached . Additionally, confidentiality was reassured.  d. Data collection period  We avoided a rushed or short data collection periods/reminders by giving a reasonable time period of about 2 weeks for each respondent , beyond which reminders via personal telephone calls/ chat messages were given.  . | These points on non-response bias were further clarified under Section 2.3 Data collection |
|  | Knowledge Assessment: The article mentions that GPs displayed lower scores in knowledge levels. Could the authors elaborate on the specific areas where knowledge gaps were observed? Were these gaps adequately addressed in the discussion and recommendations section of the article? |  | We have responded in further detail as according to Reviewer 2 comments for this section. |
|  | PeKa B40 Scheme: While the study associates positive attitudes towards the PeKa B40 scheme with acceptance and participation in the NHFS, it would be helpful to have a deeper understanding of the scheme itself. Can the authors provide more information about the PeKa B40 scheme, its objectives, and the specific aspects that need improvement based on the study findings? | Launched on the 28th January 2019 by the Hon Minister of Health Datuk Seri Dr Dzulkefly Ahmad.  Health screening PPP programme  involving public health clinics (KKs)  & private GPs  Early detection of non-infectious diseases which are more frequent in the B40 community > 40 yrs old  Managed by ProtectHealth, a non- profit subsidiary of MOH  B40 –median household income of < RM 3K  Benefits:  Screening for NCDs  Financial support for purchase of medical devices,  Cancer treatment  Transportation for hospital care  Role of GPs in PEKA B40 – Screening only  GP dissatisfaction:  Low consultation remuneration  1st visit RM 40  2nd visit RM 20  Superficial work scope | This has been further elaborated in pg 17 of the revised manuscript. |
|  | Generalizability: Given that the study focused on private GPs in Malaysia, to what extent can the findings be generalized to other healthcare contexts or different types of healthcare providers? Are there any limitations in the study design or sample selection that may affect the external validity of the results? | . | External validity examines whether the study findings can be generalized to other contexts. This study focused on private Malaysian GPs on the specific areas described. Other healthcare workers with closely related but quite distinct working environment would be their public primary care counterparts in the government health clinics. There is a possibility of the knowledge and attitude components be similar in findings, except with respect to PeKa B40 involvement. In this case, the roles of public clinic doctors in PeKa B40 are broader and deeper in coverage. Additionally, the other limitation would the geographical distribution of the public clinics which are far less than the number of GP clinics and may not allow sufficient random stratification into urban and rural samples.  On a lesser scale, the findings may be generalised to community pharmacists but again with the exception of the PeKa 40 issues and gatekeeping function. |
|  | Practical Implications: The article suggests that the results of the study should be strongly considered by the government. Can the authors provide more specific recommendations for policymakers and stakeholders based on their findings? How feasible are these recommendations in the current healthcare system? | . | On the issue of PeKa B40, the immediate steps are to allow a full management of the patients whom they detected during the screening procedures, revise the reimbursement to a more acceptable rate that commensurate with the work involved and finally, to simplify the administrative paperwork. This response is feasible under the current healthcare system.  Although gatekeeping has been practised in the public clinics to a large extent, it is yet to be formalised for the private GPs. But GPs serving on the panels of corporations or factories in the majority of cases may have to exercise some gatekeeping processes as required by these institutions. Otherwise , until there is a national health finance scheme with only one centralised payer and formal legalisation of the gatekeeper roles, private GPs will be subjected to commercial market forces under the current healthcare provision |
| **Reviewer #2** | In the background section, the references to Malaysian studies lack depth. Would have been better to cite references from more recent studies concerning national health financing schemes in Malaysia. How would you define the role of “gatekeeping” that aligns with the objectives of your study? The premise set for the role of the private GPs as “gatekeepers” in health financing schemes is insufficient as to how their contribution can better the primary health care system of Malaysia. To elaborate more on this you may cite the following Malaysian studies:  • PMCID: PMC8064717  • PMCID: PMC6510413 | Willems, D.L. 2001. Balancing rationalities: gatekeeping in health care. J Med Ethics. Feb;27(1):25-9.    PMCID: PMC8064717  Mubarak N, Raja SA, Khan AS, Kanwal S, Saif-Ur-Rehman N, Aziz MM, Hussain I, Hatah E, Zin CS. A Conceptual Framework of the Way Forward to a Community Pharmacist-General Practitioner Collaborative Medication Therapy Management Model for Chronic Diseases in Malaysian Primary Care: A Qualitative Study. Risk Manag Healthc Policy. 2021 Apr 19;14:1615-1627. doi: 10.2147/RMHP.S296113. PMID: 33907479; PMCID: PMC8064717.  .. | These 2 specific articles are added to specifically answer these 2 questions.  All other added articles are as described earlier. |
|  | Instead of mentioning the development of various national health schemes, the focus would have relied on schemes tailoring specifically to the needs of private GPs to support the rationale of your study. | healthdirect .What is Medicare? [cited 2023 July 11] Available from: <https://www.healthdirect.gov.au/what-is-medicare>  Jolyon Attwooll. Government flags shift in fee-for-service model.RACGP. 2023. Jan 23. [cited 2023 July 11]. Available from: https://www1.racgp.org.au/newsgp/professional/government-flags-shift-in-fee-for-service-model  Thailand’s Universal Health Coverage- Regional Seminar “Rethinking Social Protection in a Changing Arab Region” Amman, Jordan 13 -15 May 2014 Dr. Thaworn Sakunphanit MD., FRCPT, BA (Econ), MSc. (Social Policy Financing) Director, Health Insurance System Research Office (HISRO).. https://www.ilo.org/wcmsp5/groups/public/---arabstates/---ro-beirut/documents/genericdocument/wcms_246594.pdf | These 3 articles were added in response to the said comments. |
|  | Please provide more insights into the PeKa B40 health financing scheme with a compelling rationale to support its inclusion in national health financing schemes. How it can facilitate private GPs in improving the primary healthcare structure of Malaysia? Since PeKa B40 is a pilot public-private partnership program, why the study incorporated its use by private GPs only? |  | Same response to Reviewer 1 |
|  | In questionnaire development, the authors state: “The self-designed questionnaires involved the contents from the literature review” However, no references have been cited to provide details of the literature review. Please provide details for the development of the questionnaire (how many statements were added/removed/modified) in the idea generation phase. | Examples of questions developed from the literature review as follows:  **A.Knowledge of health financing schemes**  1.Tax-based finance refers to financing resources derived from government revenues  (Savedoff, W. 2004. Tax-Based Financing for Health Systems: Options and Experiences. Discussion Paper No.4.World Health Organisation)  2. In SHI, payment models can be either single or multi-payor systems.  (Abrejo, F.G. & Shaikh, B.T. 2008. Social health insurance: can we ever make a case for Pakistan? J Pak Med Assoc. May;58(5):267-70)  3. Private insurance is not the preferred choice for Universal Health Coverage  (Duggal, R. 2011. Private health insurance and access to healthcare. Indian J Med Ethics. 8(1):28-30).  **B. Knowledge of gatekeeper**  1. A GP gatekeeper triages the patient's further access to the health system.  (Willems, D.L. 2001. Balancing rationalities: gatekeeping in health care. J Med Ethics. Feb;27(1):25-9)  **C. Knowledge of PeKa B40**  1. The PEKA B40 scheme covers those from the minimum age of 40 years old.  (PeKa B40 https://protecthealth.com.my/peka-b40.html)  In relation to the questionnaire development, they are self-administered because they are cost-effective, easy to administer for small and large groups and self-paced. They are close-ended for nominal and ordinal variables. Likert scales are adopted. Open-ended questions are limited to some responses from PeKa B40 providers *eg How many patients have you signed up since registration?*  In the idea generation phase, a pilot study of the survey questions involving 30 GP respondents by convenient sampling provided no major flaws. Additionally, via the Smart PLS protocol, the questionnaires passed the convergent and discriminant validity as well as multicollinearity analysis.  Hence there were no modification nor deletion of the survey questionnaires. | These articles were added in Section 2.2 on Questionnaire development |
|  | In the demographics section for the nature of the practice of private GPs, please clarify what you mean by “group practices” here. With reference to the “ownership” of private GPs please clarify what you mean by Partner, Director, and Employee. Do these correspond to the private GPs in university-based settings or private clinics? | Group practices are typically divided into single-specialty and multispecialty practices. In this case, being GPs, they are of the single specialty type. The defining characteristic of single-specialty group practice is the presence of two or more GPs providing patients with one specific type of care ie primary care  .  Employee refers to GPs being employed within one of several practice models. In this case, the GP is employed according to the local labour laws.  Unlike salaried doctors, GP partners own a share of their practice and draw income from business profits.  As a partner, one is responsible for the running of the practice and will be expected not only to provide clinical sessions, but also to play an active role in the administrative and business side of the practice. Unlike salaried GPs, there is no set income range for GP Partners.  As directors, the clinic practice is structured along  corporate lines. Hence, each director owns ownership by the number of shares and the finances and working structure is guided by the Companies Act. Subsequently, apart from being working directors, they can also employ other doctors to work in their group practice.  The above categories in the study are all referring to the Malaysian private GP settings and not in the university-based settings. | These statements are added in the Section on Results 3A Descriptive statistics in pages 25 and 26 of the revised manuscript. |
|  | In the discussion section, please state the knowledge, attitude, practice, and participation aspects of your study separately and explicitly with further mentioning the areas of improvement in knowledge, attitude, and practice areas. Requires mentioning of comparisons of health financing schemes in other low-middle income countries. Please mention the implications and future prospects of your study | **a.Knowledge:**  By Bloom’s scoring classification, good knowledge of the health finance system, gatekeeper roles, and PeKa B40 are noted at 17.6%, 44.6%, and 27.0%, respectively. Malaysian GPs are very knowledgeable about their gatekeeper roles as compared to their levels of knowledge on health financing schemes and PeKa B40. This implies that the majority is already aware of this role they should be playing, although the policy has yet to be enforced. The GPs will be better prepared to assume this role without much difficulty. However, Malaysian GPs will need to have a more in-depth understanding of the types of health financing schemes that constitute their greatest deficit. This can be overcome by providing more information and education, namely through the internet, such as through the organisation of webinars and other interactive courses, as these channels are the most popular sources of information, apart from the mass and social media.  The emphasis on lack of knowledge of health financing schemes has been similarly observed in a Kenyan study by Mathaeur et al in 2008. 19 focus-group discussions of various groups in the informal sector has been done with the objective to analyse and understand the demand for social health insurance by informal sector workers in Kenya. The perceptions and knowledge of and concerns regarding social health insurance have been assessed. The conclusion is that the lack of knowledge about the NHIF (Kenyan National Hospital Insurance Fund) and its enrolment processes, has formed the most critical barrier. [33]    **b.Attitude**  Bloom’s scores indicating good attitude is seen most strongly towards gatekeeper roles at 36.5%, followed by PeKa B40 at 18.2% and health finance schemes at 16.2%.  The high scores on attitude towards gatekeeper roles synergises with their relatively high knowledge scores on the same area. The majority of respondents agree that GPs shall assume the full gatekeeper function. This indicates their strong willingness and readiness to execute this duty.  In a recent Indonesian study however, the scores on attitude towards the gatekeeper role by Indonesian GPs has attained lower values than knowledge. And the low level of attitude has influenced their performance. [34] This is in contrast to the findings of our study, in which the attitude component is more significant with higher scores than the knowledge factor.  **c.Practice**  Practice matters relate to the various consultation processes in PeKa B40, which are the history, physical examination and investigation areas and each of their components. Overall Blooms’ scores of 64.9% in the good category indicates a high acceptance rate to the inclusion of these processes.  **d.PeKa B40 Provider Status**  Although the Peka B40 program is a good initiative of the government to initiate the public-private primary care partnership involving Malaysian GPs and the various KKs, the responses by the GPs in the survey are not forthcoming. A total of 60.5% (n = 179) of respondents are non-PeKa B40 providers.  **e.Acceptance and Level of Participation in the National Health Finance Scheme.**  Bloom’s scores indicate 31.1%, 39.9% and 29% under “ good, moderate and poor” categories. The high moderate scores may partly reflect the lack of knowledge in health financing schemes and the low acceptance of the PeKa B40 scheme. Effective measures to resolve these issues may be able to result in a higher migration from the moderate to the good categories. However, a combined score of 71% for the good and moderate categories currently indicates a significant acceptance and participation level in the NHFS.  **f.Gatekeeper**  As the first point of contact in the community. the GP needs to understand that the gatekeeper role requires a delicate balance between giving the best of medical care for his patient as opposed to the cost control measures. His professional competency as a primary care physician managing his patient may reach a point where referrals for secondary and tertiary specialty care will be necessary over and above the issue of trying to reduce healthcare costs. That important judgemental skill has to be diligently observed whilst maintaining the patient’s trust.  The fact that gatekeeping is already being practised in the government clinics in Malaysia, is all the more justification that GPs must be able to do the same. This will help to reduce inequalities and expand integrated care.  Hence, gatekeeping needed also to be introduced into the private sector. With regard to integrated care, the benefits of the gatekeeping function will also spill over to other allied health care givers. To begin with, this includes specific collaborative models in chronic disease management with community pharmacists. GP-led integrated teams are the way forward for primary care delivery.  **g.National health financing scheme**  Recent events in Malaysia have given a very strong indication on the Malaysian government’s commitment to transform the healthcare system and implement a national health financing scheme (NHFS). The 1Care system is the last comprehensive proposal that has a hybrid structure of a tax-based and social health insurance schemes. The reform will significantly result in a better coverage and positive outcomes which will be observable in the current situation. Unfortunately, the government of the day has to give in to opposition voices objecting to the social health insurance component. Worse still, one of the strongest opposition comes from the medical professionals, including the GPs and other secondary and tertiary clinicians, albeit for their own different reasons. This may be attributable to the lack of knowledge of social health insurance and the misconception of the gatekeeper roles amongst the doctors. Political rivalries further aggravate the issue.  However, the growing bipartisan movement to rejuvenate the NHFS recently, is a positive sign for Malaysia. But in the move to achieve this, it must be borne in mind that universal health coverage requires an effective structuring and management of health financing. Of utmost importance is a need for good governance and leadership so as to reduce inefficiencies and wastages of the various funds and resources. Without doubt, a strong primary care is required to achieving universal health coverage. The duality of public-private healthcare services in Malaysia needs to be well integrated at all levels of care.  One of the most relevant and successful models for the country to emulate comes from its nearest neighbour, that is Thailand. The country has ensured complete UHC for all segments of the population with a hybrid tax-based and social health insurance schemes. Additionally, the purchaser-provider split and gatekeeping at the primary care level, together with a comprehensive benefits package, has resulted in a highly successful structure. Underlying this is the fact that the support and collaboration between their healthcare professionals, civil societies and politicians has been crucial in ensuring its success.  **Implications & future prospects of the study:**  **Theoretical Contributions:**  As this was an original nationwide study and the model was proven to fit reasonably well, as confirmed by the R squared value, the questionnaires in the survey and other aspects of the study design could be a platform for future studies with added refinements. Due to the scarcity of research materials on issues concerning private Malaysian GPs, this study will be able to stimulate more of such activities.  **Practical Contributions:**  The results of this study should be strongly considered by the government in formalising the policy in defence of the interests of private Malaysian GPs in the NHFS. This will help to ensure the success of the NHFS when it is implemented.  **Future prospects of study:**  There are many more issues regarding private Malaysian GPs that require further research. These include public-private partnerships between government primary care doctors in community clinics (Klinik Kesihatan) and private GPs, case-mix and costings in primary care as well healthcare delivery by family physician-led multidisciplinary primary care teams. | These statements were added in the Discussion section as advised. |
